# The Nanostructuring of Atomically Flat Ru(0001) upon Oxidation and Reduction

**DOI:** 10.1186/s11671-016-1757-2

**Published:** 2016-12-01

**Authors:** A. Goriachko, H. Over

**Affiliations:** 1 Department of Physical Electronics, Taras Shevchenko National University of Kyiv, Glushkova 4G, Kyiv, 03022 Ukraine; 2 Department of Physical Chemistry, Justus Liebig University, Heinrich Buff Ring 17, 35392 Giessen, Germany

**Keywords:** Ruthenium, Surface, Oxidation, Reduction, Scanning tunneling microscopy

## Abstract

The O/Ru(0001) system is widely studied due to its rich phase variety of various stoichiometry and atomic arrangements, including the formation of a RuO_2_/Ru(0001) oxide layer. Apart from homogeneous ruthenium surfaces in certain oxidation states, also strongly heterogeneous surfaces can exist due to oxidation state’s variation at the nanoscale. We report on a scanning tunneling microscopy (STM) study of the nanostructuring of the oxidized Ru(0001) surface as a result of its interaction with molecular oxygen at elevated temperatures and subsequent reduction of a resulting RuO_2_ film by CO or HCl molecules from the gas phase in high-vacuum environment.

## Background

Ruthenium is an important material in modern catalysis, as recently reviewed in [1], where the catalytically active phase has shown to consist of a surface layer of ruthenium dioxide. The latter is electronically conducting thus being employed as electrode material and contact material in super caps [1]. Another interesting aspect of ruthenium is the ease of its (0001) crystal face to support two-dimensional (2D) materials in a laterally nanostructured state, as demonstrated for boron nitride [2], graphene [3], and their mixtures [4]. These systems can act as nanotemplates for the growth of various catalytically active metal nanoparticles [5, 6], while the preparation of the 2D/Ru(0001) nanotemplate itself is strongly dependent on the previous preparation of the ruthenium sample in molecular oxygen at elevated temperatures. This “technological” step drives carbon impurities (always present in bulk Ru) out of the subsurface region toward the surface thus strongly affecting the resulting stoichiometry within the 2D B_x_N_y_C_z_ mixture on top of Ru(0001).

The oxygen treatment of Ru(0001) usually starts with the ordered oxygen overlayer on top of the metal surface without oxygen penetration below the surface. As the oxygen coverage increases, the adsorbed O atoms form a 2 × 2 superstructure at 0.25 ML (monolayer) and further a 2 × 1 superstructure at 0.5 ML [7, 8]. Toward 1 ML coverage, there appears a (2 × 2)-3O [9, 10] and finally a simple (1 × 1)-O overlayer [11]. At even higher oxygen exposures and elevated temperatures of around 650 K, the Ru(0001) surface reveals a self-limiting growth of a 1.6-nm thick heteroepitaxial RuO_2_(110) film [12].

The RuO_2_(110)/Ru(0001) system was investigated by means of scanning tunneling microscopy (STM) and ab initio density functional theory (DFT) calculations enabling its exact atomic structure clarification [13]. Such kind of surface exposes parallel rows of Ru atoms and so-called “bridge” O atoms. The RuO_2_(110) surface is an efficient catalyst for CO oxidation even at temperatures as low as 300 K [14]. There is evidence that the RuO_2_(110)/Ru(0001) system can be laterally inhomogeneous, as observed for instance by low-energy electron microscopy (LEEM) in the form of (1 × 1)-O in coexistence with RuO_2_(100) and RuO_2_(110) [15, 16]. However, due to insufficient lateral resolution of the LEEM technique, the nanoscale structure of the RuO_2_(110) could not be fully resolved. Therefore, the STM technique was applied to study the strongly inhomogeneous RuO_2_(110) films, such as at initial nucleation stage [17–19] or after partial thermal decomposition of the oxide [20].

Despite some nanoscopic insights into the RuO_2_(110) growth on Ru(0001) already documented in the literature, our view on this process is still far from being complete. There is a certain knowledge gap at one of the most interesting stages, roughly halfway in between the nucleation onset and the final continuous state of the oxide film. Clearly, at this stage, a pronounced nanostructuring of the surface can be observed without resorting to any kind of time-consuming nanolithography techniques thus being potentially interesting for a range of applications, e.g., nanocatalysis, nanobiotechnology, and nanoelectronics. In the present study, we report on the nanostructuring of the RuO_2_(110)/Ru(0001) system and its extensive characterization employing STM and X-ray photoelectron spectroscopy (XPS). In the following, we have chosen a rather narrow oxidation temperature range of 600–650 K, as a trade-off between requirements not to drive the system into a spatially homogeneous equilibrium state and to maintain a high enough mobility of the species for effective oxidation.

## Methods

All experiments were conducted in a home-built ultra-high vacuum (UHV) multi-chamber system with a background pressure in the lower 10^−10^ mbar range. The initial preparation of the single crystal Ru(0001) surface (sample size 5 mm × 5 mm × 5 mm, delivered by Mateck) always included sputtering with 1.5 keV Ar^+^ ions (Ar purity of 99.999%, delivered by Linde), the sample being held at 1100 K for continuous healing of the ion beam-induced damages of the crystalline structure of ruthenium. An undesired side effect of ion sputtering is the departure from the uniformly smooth surface if viewed at the micrometer scale. Then, the general specifics of the sample are the coexistence of extended atomically flat terraces with abrupt height changes of several dozen nanometers (essentially step-bunching areas). A previous photoelectron spectromicroscopy investigation has demonstrated a substantially faster oxidation kinetics of such step-bunching areas in comparison with atomically flat ones [21], later directly confirmed on the atomic scale by the STM technique [17].

The ion sputtering was followed by exposing the surface to molecular oxygen (purity 99.999%, delivered by Linde) at pressures in the 10^−8^–10^−7^ mbar range for several dozen minutes and the sample temperature kept at ~1100 K. Such a treatment typically removed carbon contamination from the near-surface region of the sample: carbon segregates from the bulk/selvedge region of the sample to the surface and is subsequently converted into gas phase CO or CO_2_ molecules. The RuO_2_ growth (oxidation) was performed during several dozen minutes at elevated sample temperatures (600–650 K) in molecular oxygen at constant pressure in the 10^−5^–10^−4^ mbar range. Afterwards, RuO_2_ can partially be reduced by interaction with reducing molecules such as CO (purity 99.999%, delivered by Linde) or HCl (purity 99.9%, delivered by Linde) at typical pressures of 10^−7^–10^−8^ mbar.

The surface analysis was performed using the XPS setup delivered by PSP Vacuum Technology. It features a dual X-ray source (Mg Kα radiation hν = 1253.6 eV, 200 W power) together with a hemispherical analyzer (20 eV constant pass energy). The surface topography of the samples after various treatments described above was investigated in situ by means of STM in constant current mode (VT-STM, delivered by Omicron). All measurements were performed at the background pressure in the UHV range and always after the sample has cooled to room temperature. We have used metallic probe tips hand cut from the Pt_80%_Ir_20%_ Ø 0.25 mm wire (purity 99.9%, delivered by Sigma Aldrich).

## Results and Discussion

**Fig. 1:**
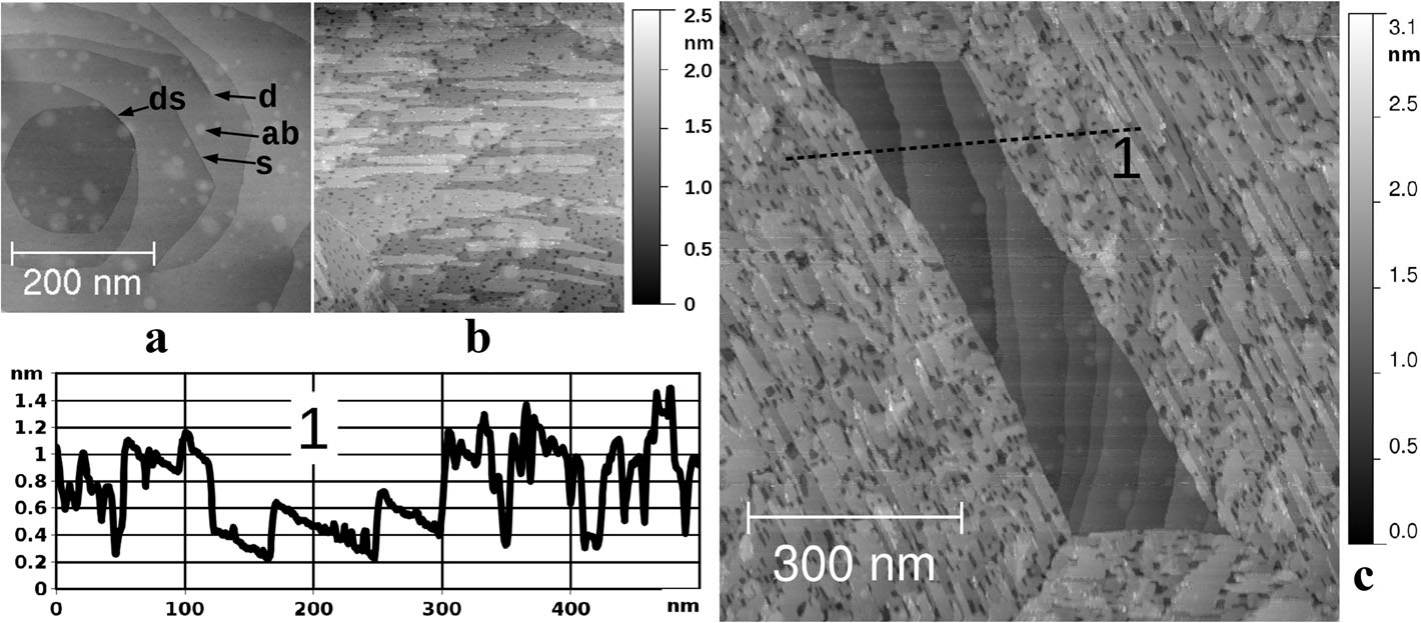
STM images (sample bias voltage 1.0 V; tunneling current 1 nA) of the single crystal Ru(0001). **a** Prepared according to the ion bombardment protocol (see Methods) followed by etching at 1100 K in O_2_ at 5 × 10^−8^ mbar during 30 min. **b** The same as **a** but with additional oxidation at 630 K in O_2_ at 3 × 10^−5^ mbar during 50 min. Both images (**a**, **b**) have a visible area of 430 nm × 430 nm and share the same gray scale ruler shown right next to them. **c** The same as **a** but with additional oxidation at 630 K in O_2_ at 3 × 10^−5^ mbar during 40 min; visible area 860 nm × 860 nm, a height–distance cross-section along the *black dashed line 1* is given to the left of the image

First, we survey the surface morphology of Ru(0001) with and without the oxide film. Throughout this work, we consistently present only those locations on our sample, which did not include any step-bunching regions. The STM images (430 nm × 430 nm) of “metallic” and “oxidic” morphologies are presented in Fig. 1a–b. In Fig. 1a, we observe a typical Ru(0001) surface, exposing terraces separated mostly by single atomic steps “s” or in some cases double atomic steps “ds”; also visible are the screw dislocations “d” coming out at the surface. On the top of atomically flat terraces, we notice irregularly placed and shaped elevations “ab”, which closely resemble elevations above the buried argon bubbles after similar preparation of Ru(0001) reported by Jakob et al. [22] to be stable up to 1200 K. However, we could not detect the Ar 2p emission by means of XPS, as the total Ar concentration is most probably not high enough to reach above the noise level of our measurement. Nevertheless, we tentatively associate the “ab” type features with argon bubbles trapped below the surface as a result of Ar^+^ ion sputtering preparation step. It is worth noting that other impurities of relevance to our experiments produce different types of morphological features on the Ru(0001) surface, as demonstrated for carbon in [3, 4] and for oxygen below. In Fig. 1b, we show a typical surface of the RuO_2_(110) film, consisting of numerous atomically flat terraces, single atomic steps, and holes with lateral extensions in the 10 nm range. Clearly, a nanostructured surface is created in this case as a result of the oxidation process.

Figure 1c presents an STM image of the 860 nm × 860 nm surface area of the partially oxidized sample with RuO_2_(110) present on the periphery and non-oxidized atomically flat Ru(0001) in the center (parallelogram-shaped patch). This morphology is in line with the corresponding LEEM results, showing the growth of RuO_2_(110) domains with three distinct orientations along the high symmetry directions of the Ru(0001) surface [15, 16]. The parallelograms can arise when the surface is not yet completely covered by the oxide and four domains of two different orientations (in alternating order) form a closed contour around an internal metallic patch.

A cross-section along the black dashed line in Fig. 1c is given in the lower left part of the figure showing the height jump of ~0.6 nm between the oxide and the metal. This value is not identical to the oxide film thickness because the oxygen penetration into the ruthenium during the growth process will shift the oxide/metal interface below the unreacted metal surface. Another interesting feature, that can be deduced from this cross-section is that the basements of the holes within the oxide film often reach down to the non-oxidized Ru(0001). This means that such holes are essentially nanoscale perforations of the RuO_2_(110) film; thus, the overall surface is strongly heterogeneous (metallic vs oxidic) both at the micro and the nanoscale.

**Fig. 2:**
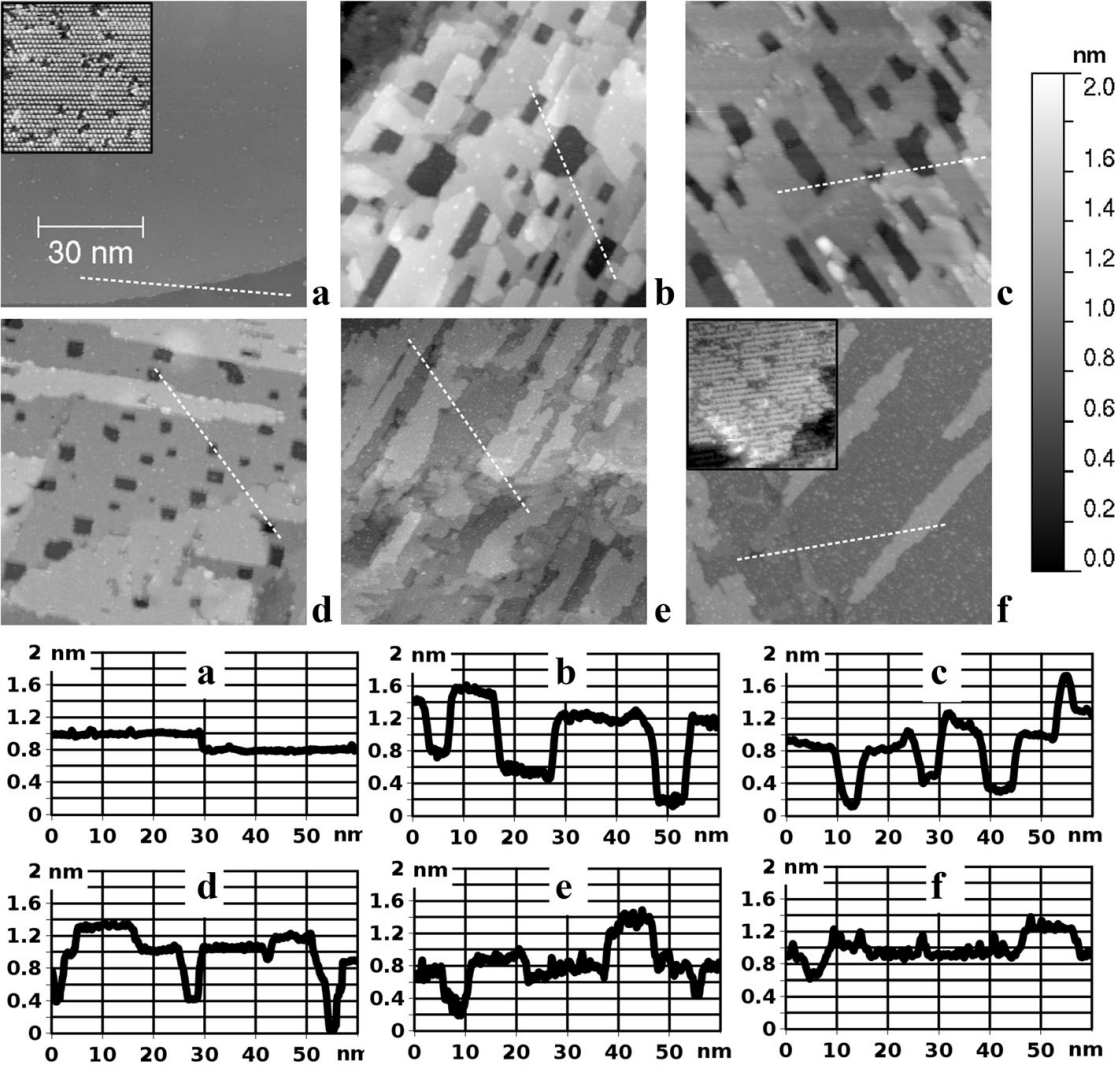
STM images (86 nm × 86 nm, sample bias voltage 1.0 V, tunneling current 1 nA) of the single crystal Ru(0001) sample at different stages of oxide film growth. **a** The initial atomically flat metallic substrate; **b**–**f** the oxide film grown at 630 K in O_2_ at 3 × 10^−5^ mbar during **b** 30 min, **c** 40 min, **d** 50 min, **e** 1 h, and **f** 1.5 h. All images share the same gray scale ruler given to the right. The height–distance cross-sections along the dashed lines are presented in the lower part of the figure. The insets in **a** and **f** are 17.2 nm × 17.2 nm images with atomic resolution

Next, we study the various stages of oxide growth, which are summarized in Fig. 2, starting from the initial atomically flat metallic substrate (a) followed by the isolated oxide patches (b–d) and finally the formation of a continuous oxide film (e, f). The depicted sequence represents essentially a film growth process with a simultaneous consumption of substrate material. It starts on a metallic substrate with atomically flat terraces separated by single atomic steps (Fig. 2a), when there are only O atoms on the surface in the form of the 2 × 2 phase. This is the lowest coverage oxygen phase on Ru(0001) with a total coverage of 0.25 ML and a lateral distance between neighboring O atoms equals to 0.54 nm [7, 23], being resolved in the 17.2 nm × 17.2 nm STM image (see inset in Fig. 2a).

When the oxide film appears on top of the substrate (Fig. 2b), it has nanoscale perforations, as already discussed in Fig. 1b, c. As the amount of oxide increases, the perforations become smaller in lateral dimensions (Fig. 2b–d). At this growth stage, the film is essentially nanostructured in the form of apertures up to ~10 nm in lateral size and up to ~1 nm in depth. Finally, the perforations vanish, and the film becomes continuous (Fig. 2e). At the final growth stage, there is a smoothening of the surface, leading to the formation of extended atomically flat RuO_2_(110) terraces (Fig. 2f), and the film is no longer nanostructured. The corresponding atomic scale STM image is presented in the inset in Fig. 2f. Visible here are rows of the so-called bridging O atoms, which are the topmost atoms of the given stoichiometric RuO_2_(110) surface [13]. These parallel rows run along the [001] direction at a distance of 0.64 nm and are clearly resolved in the image. The neighboring O atoms within a row are only 0.31 nm apart and are barely resolved as the atomic corrugation along the rows is roughly at the noise level of our measurement.

**Fig. 3:**
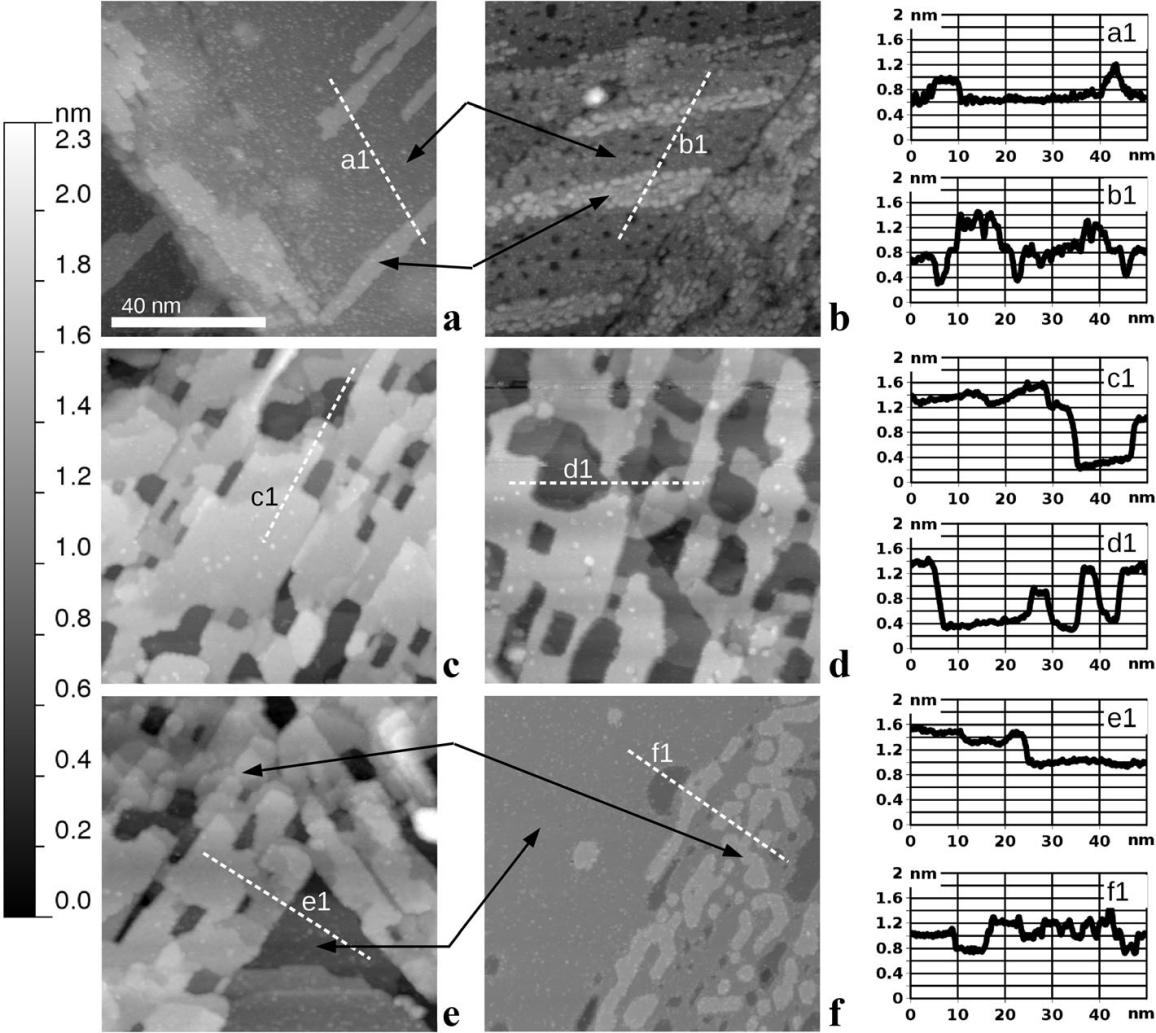
STM images (86 nm × 86 nm, sample bias voltage 1.0 V, tunneling current 1 nA) of the RuO_2_(110)/Ru(0001) sample before (**a**, **c**, **e**) and after (**b**, **d**, **f**) being chemically reduced: **b**, **d** after exposure to 10 L (1 × 10^−7^ mbar for 100 s) of CO, while the sample was held at 600 K; **f** after exposure to 100 L (1 × 10^−6^ mbar for 100 s) of CO, while the sample was held at 600 K. All images share the same gray scale ruler given to the left. The height–distance cross-sections along the *dashed lines* are presented in the rightmost column of the figure

Provided the oxide film is present on the surface, we focus now on the changes of the film’s structure (particularly nanostructure) induced by interaction with a reducing agent (carbon monoxide (CO)). In Fig. 3, the STM images in the left column (a, c, e) depict the initial state of the oxide film and those in the right column (b, d, f) the final state after the complete or partial oxide reduction. In Fig. 3a, we observe a smooth and continuous RuO_2_(110) similar to that in Fig. 2f, while shown in Fig. 3b is the same sample after exposure to 10 L of CO at 600 K. The result of CO exposure is again a nanostructuring of the initially smooth surface, namely, some shallow (not through the entire film) pits with lateral sizes in the nanometer range that are etched out amidst the formerly flat areas. Also, the topmost narrow terraces are completely converted to aggregates of metallic nanoclusters, as they are attacked by CO molecules at their perimeter, and the reduction reaction spreads parallel to the surface. Shown by black arrows is the correspondence between different types of areas before and afer the reduction.

In Fig. 3c, the initial state is non-continuous (nanoperforated film), and the result of a similar exposure (10 L CO at 600 K) is shown in Fig. 3d. Now, the major effect is lateral widening of the holes (some of them now reaching several dozen nanometers); in other words, we observe lateral etching, which proceeds isotropically in the surface plane. Much higher exposure (100 L CO at 600 K) had an effect of complete oxide film reduction, as demonstrated by the sequence from Fig. 3e to Fig. 3f. The latter STM image shows the surface with numerous single atomic layer islands and pits of several nanometers in their lateral size, observed in the right part of Fig. 3f. However, in the left part of Fig. 3f, we observe the same morphology and structure as in Fig. 2a, indicating the presence of the oxide-free patch prior to reduction. It seems like a natural explanation that the exact area imaged in Fig. 3f consists of both an oxide-covered patch (right) and oxide-free patch (left) prior to reduction. Again, the pairs of black arrows express the correspondence between these two types of areas before and after the reaction with CO. The border between these two patches is a section of the parallelogram’s perimeter (of the one similar as in Fig. 1c or the one partially imaged in Fig. 3e). If viewed on atomic scale, both types of areas in Fig. 3f show the same 2 × 2 superstructure as the inset in Fig. 2a.

**Fig. 4:**
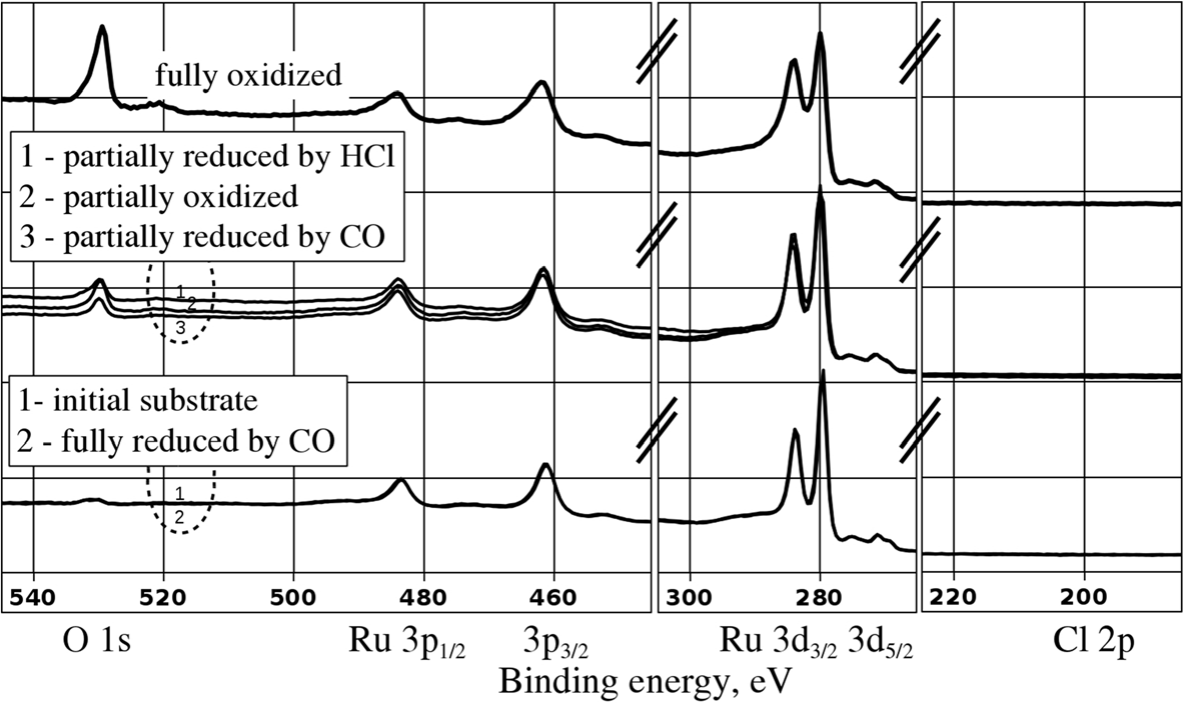
XPS spectra of the Ru(0001) after various chemical treatments. The spectra were logically grouped by means of shifting along a vertical axis (intensity in arbitrary units). The group of two curves in the lower part of the plot contains spectra of the sample before oxidation (*initial substrate*) and after full reduction of the previously existing oxide film (*fully reduced by CO*). The group of three curves in the middle part of the plot contains spectra of the surface covered by non-continuous oxide film as a result of oxide growth (*partially oxidized*) and reduction of the previously existing oxide film (*partially reduced by HCl*, *partially reduced by CO*). A single curve in the upper part of the plot (*fully oxidized*) is the spectrum obtained on the Ru(0001) sample, which was oxidized to saturation

The STM data presented above provide an in-depth characterization of the surface morphology after certain well-defined chemical treatments of the Ru(0001) substrate. In order to verify the elemental composition of the surface, we performed the XPS characterization of our sample after selected preparation protocols. In Fig. 4, we present the XPS data gathered on the initial ruthenium substrate, after its partial or full oxidation, as well as partial or full reduction. Visible are the spectral features of the Ru 3p1/2, 3p3/2, 3d3/2, 3d5/2, and O 1 s emissions. In the lowest group of two spectra, the “initial substrate” spectrum was taken on the surface shown in Fig. 2a. Apart from ruthenium peaks, we also observe a very weak emission in the O 1 s region, corresponding to the 0.25 ML of O atoms chemisorbed on top of ruthenium (2 × 2 superstructure, see inset in Fig. 2a).

In the middle group of curves, a spectrum code-named as “partially oxidized” was obtained on the sample shown in Fig. 2b. Here, the O 1 s emission is present in the spectrum at the level typical for the non-continuous (nanoperforated) RuO_2_(110) film. The sample with a continuous atomically flat oxide film (shown in Fig. 2f) produced a photoelectron spectrum shown by the upper curve in Fig. 4, designated as “fully oxidized”. Its hallmark is the O 1 s emission of the intensity higher than the Ru 3p peaks and comparable with those from the Ru 3d core levels.

Further, we have acquired the XPS spectrum after partial reduction of the oxide film by CO, designated as “partially reduced by CO” in the middle group of curves in Fig. 4 and corresponding to the surface depicted in Fig. 3d. Here, the O 1 s emission has a similar intensity as that from the as-grown “partially oxidized” sample, consistent with the incomplete coverage of the surface by the oxide in the corresponding STM images. Finally, the spectrum obtained after complete reduction of the oxide in CO can be found in the lower group of curves (marked “fully reduced by CO”). Notably, this spectrum is basically identical to the “initial substrate” in the lower group of curves. It was obtained on the surface shown in Fig. 3f thus completing the full circle: metallic surface–metal partially covered by oxide–metal fully covered by oxide–metal partially covered by oxide-metallic surface. Under the “metallic surface,” we mean the Ru(0001) surface with chemisorbed 0.25 ML of oxygen forming the 2 × 2 superstructure. The return to this surface condition is evidenced by STM (Fig. 3f) and identical O 1 s emissions in the lower group of curves in Fig. 4. Overall, we observe an excellent correlation between the intensity of the O 1 s emission and the amount of oxide-related morphological features on the surface of our Ru(0001) sample.

Finally, we compare the above described reduction process induced by CO with that caused by interaction with HCl molecules. For this purpose, we have exposed the sample shown in Fig. 2e to 10 L (1 × 10^−7^ mbar for 100 s) HCl at substrate temperature of 700 K. The corresponding XPS spectrum is marked as “partially reduced by HCl” in the middle group of curves in Fig. 4. It is worth noting that no Cl 2p emission is visible in the spectrum after partial reduction by HCl. A separate analysis has indicated that this emission is actually present but its intensity is very small. Thus, we can neglect the presence of chlorine on the surface, which stands in line with its release into the gas phase during the RuO_2_ reduction above 650 K [24].

**Fig. 5:**
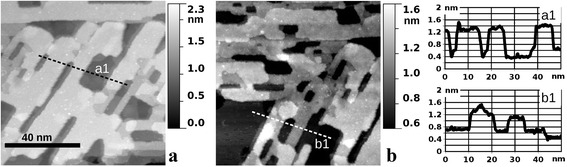
STM images (86 nm × 86 nm, sample bias voltage 1.0 V, tunneling current 1 nA) of the RuO_2_(110)/Ru(0001) sample after being chemically reduced by exposure to 10 L (1 × 10^−7^ mbar for 100 s) of HCl, while the sample was held at 700 K. **a** An area which was covered by the oxide homogeneously before the reduction. **b** An area which partially contains the parallelogram-shaped metallic patch prior to reduction

The STM images of the oxide film partially reduced by HCl are depicted in Fig. 5. Obviously, the initially continuous film (Fig. 2e) becomes nanostructured in the form of nanoperforations. In Fig. 5a, the perforations are different from the cases of as-grown or CO-etched oxide by an apparent elongated shape in the direction coincident with the rows of bridging O atoms. This is an indication of the preferential sidewall etching of the RuO_2_ grains along the [001] direction or its equivalents. Since such directions are parallel to the sides of the “parallelograms” (as in Fig. 1c), it is natural to expect their preservation during the reduction process. Indeed, such parallelogram-shaped extended oxide-free areas were found on the given sample, and one of them is partially imaged in Fig. 5b. In this STM image, the gray scale is specially tuned to the height of the remaining RuO_2_ surface. The latter consists of atomically flat terraces, similar to that after partial reduction by CO, with [001] rows of bridging O atoms still recognizable although with a noticeable degree of disorder on the atomic scale. The major difference between the interaction of RuO_2_(110)/Ru(0001) and both reducing agents (CO and HCl) is thus the laterally isotropic vs anisotropic spreading of the reaction front. One possible explanation is a higher mobility of the chlorine ions along the [001] direction. Further investigations will be required in order to obtain a complete picture of RuO_2_ reduction by HCl, including the initial stages as well as complete reduction and their corresponding surface morphologies.

## Conclusions

In conclusion, we have identified by means of STM investigation a variety of Ru(0001) oxidation and RuO_2_(110)/Ru(0001) reduction processes leading to nanostructured (and/or heterogeneous) surfaces when started from atomically smooth morphology of the chemically homogeneous sample. The oxide growth at 650 K in O_2_ proceeds through the nanostructured stage later reaching an atomically flat surface of the saturated RuO_2_(110) film. The nanostructuring appears in the form of irregular nanoscale perforations within the oxide layer, while their lateral sizes can be further manipulated (increased) by partial oxide reduction in CO (at 600 K) or HCl (at 700 K) gaseous environment. Our preliminary investigations demonstrate the “etching” of RuO_2_ by HCl, which proceeds anisotropically in the surface plane, as opposed to isotropic etching by CO.

## Abbreviations

STM: Scanning tunneling microscopy
UHV: Ultra-high vacuum
XPS: X-ray photoelectron spectroscopy
